# Naked mole-rats employ a normoxic escape behaviour that is altered by social interaction

**DOI:** 10.1098/rsbl.2025.0564

**Published:** 2025-11-05

**Authors:** Pareesa Lashani, Gloria Lamontagne, Karen L. Kadamani, Matthew E. Pamenter

**Affiliations:** ^1^ Department of Biology, University of Ottawa, Ottawa, Ontario, Canada

**Keywords:** hypoxia escape behaviour, activity, velocity, sociality, metabolic rate suppression

## Abstract

Escape behaviours are a common response when animals encounter hypoxic environments. Confoundingly, naked mole-rats (NMRs) experience hypoxia while sleeping in crowded colony nest chambers, from which escape may not be desirable. In isolation, individual NMRs decrease physical activity to save energy in hypoxia, but this response is absent when conspecifics are present. However, whether NMRs try to escape hypoxia is unknown, as is the impact of sociality on any hypoxic escape behaviours. We predicted that individual NMRs would try to escape from hypoxic environments, but that sociality would reduce the drive to escape. We allowed individual and paired NMRs to choose between normoxia (21% O₂) or various depths of hypoxia (3%, 7% or 11% O₂) and non-invasively recorded their activity. Surprisingly, individual NMRs exhibited a novel normoxic escape behaviour and preferred severe hypoxia (3% O_2_) to a normoxic environment. This preference was not repeated in less severe levels of hypoxia. Paired animals also preferred a hypoxic environment over normoxia, but social interactions drove an increase in movement velocity and reduced the severity of their preferred level of environmental hypoxia to 7% O_2_. Thus, NMRs choose hypoxic environments over normoxic environments and sociality impacts this behavioural choice in hypoxia.

## 1. Introduction

Most animals rely on continuous oxygen (O_2_) delivery for aerobic energy production [1]. However, there are numerous natural environments in which O_2_ availability is limited, such as in densely populated underground burrows, at high altitude or in hypoxic aquatic regions, among others [2–4]. When exposed to acute hypoxia, the initial response of many animals is to try to escape to a region with higher O_2_ availability. Indeed, there are numerous documented examples of such hypoxia escape responses across Anamalia, and particularly in hypoxia-intolerant species. For example, various aquatic species avoid hypoxic regions [5], or employ aquatic surface respiration to supplement insufficient dissolved O_2_ in water by directly breathing air at the surface [6–8]. Similarly, *Caenorabditis elegans* and *Drosophila melanogaster* larvae both actively migrate away from hypoxic regions and towards areas with greater O_2_ availability [9,10]. In mammals, no studies have been designed to directly measure escape from a hypoxic region, but hypoxic escape behaviours, including periodic bursts of activity and rearing behaviours associated with seeking escape, are common in studies of hypoxia-intolerant rodents and fish [5,11]. Conversely, hypoxia-tolerant animals have evolved to survive in hypoxic environments and often use robust metabolic rate suppression to reduce O_2_ demand and obviate the need to leave a hypoxic environment. However, no study has explored hypoxic escape behaviours in a hypoxia-tolerant mammal.

Naked mole-rats (NMRs; *Heterocephalus glaber*) are among the most hypoxia-tolerant mammals, surviving days at 8% O_2_, hours at 3% O_2_ and minutes at 0% O_2_ [12–15]. NMRs are highly social rodents that live in colonies of 50–300 animals in eastern Africa [16]. NMRs live in subterranean burrows that are approximately 1–2 m underground, made up of complex tunnel systems that can extend for several kilometres [16]. These underground burrows are poorly ventilated, which, combined with the respiration-mediated depletion of environmental O_2_ by many animals within a confined space, creates hypoxic conditions. The nest region, where most animals in the colony congregate to sleep, is likely particularly hypoxic, whereas more distant tunnel regions are relatively normoxic [17]. As a result, NMRs are thought to spend their lives in an intermittently hypoxic habitat [18–20]. To tolerate these conditions, NMRs have developed remarkable metabolic and behavioural adaptations, allowing them to remain active and conscious in hypoxia [13,18,20–23]. Indeed, unlike most small mammals, who enter a torpor-like state in severe hypoxia, NMRs must continue to stay active because O₂ availability is unlikely to increase spontaneously in their crowded nest chamber. NMRs do reduce their overall behavioural activity in acute hypoxia; however, even at 3% O₂, they remain active and continue to explore their environment [13]. Furthermore, individual animals do not employ anapyrexia by seeking colder environments in hypoxia [22], which is a common response to hypoxic environments in other small mammals that serves to reduce systemic energy demands [24–26].

Interestingly, NMRs exhibit eusociality, a rare trait among mammals, where a single queen and select males reproduce while other colony members serve as non-breeding (and pre-pubertal) workers [27]. Although previous studies exploring physiological and behavioural responses of individual NMRs to environmental hypoxia are informative, they are potentially incomplete because they have often not considered the impact of social interactions on these responses. This is an important aspect because, unlike solitary animals, NMRs exposed to hypoxia (7% O₂) in groups of two or four do not exhibit behavioural retardation [21]. This suggests that social interaction plays an important—and possibly dominant—role in determining the behavioural response of NMRs to hypoxia.

Thus, although previous studies have explored some aspects of the behavioural responses of NMRs to environmental hypoxia, important questions remain to be explored, such as whether NMRs prefer normoxic or hypoxic conditions, if they will try to escape a hypoxic environment, and if social interactions impact these responses. To address these questions, the aim of this study was to evaluate escape behaviours of individual and paired subordinate (non-breeding) NMRs exposed to various levels of environmental hypoxia. We hypothesized that NMRs would exhibit a behavioural preference for environments with higher O_2_ availability, but that sociality would alter behavioural responses to hypoxia.

## 2. Abridged methodology

Complete details about the full methodology are provided in the electronic supplementary material. Individual or pairs of subordinate NMRs were placed, unrestrained, into a custom-designed two-chamber experimental apparatus, as described previously [13]. The experimental apparatus was maintained at 30°C, and one chamber was gassed with normoxic air (21% O_2_) while the other chamber was gassed with hypoxic air (11%, 7% or 3% O_2_). Animals were allowed to behave freely, and activity was continuously monitored with an overhead camera (Basler AG, Ahrensburg, Germany). Behavioural variables were tracked using activity detection software (Ethovision XT; Noldus Information Technology, Wageningen, The Netherlands), including cumulative time, movement speed and cumulative duration of activity in each chamber and velocity. Significant differences (*p* < 0.05) were determined separately for solitary and paired animal experiments using two-way analysis of variance (ANOVA) with Holm–Sidak post-tests to assess differences between normoxia and hypoxia for each experimental variable. Differences in velocity between solitary and paired experiments were analysed with a Mann–Whitney test. Data are expressed as mean ± s.e.m.

## 3. Results

### 3.1. Individual naked mole-rats prefer severe but not moderate or mild hypoxia to normoxia

**Figure 1.**
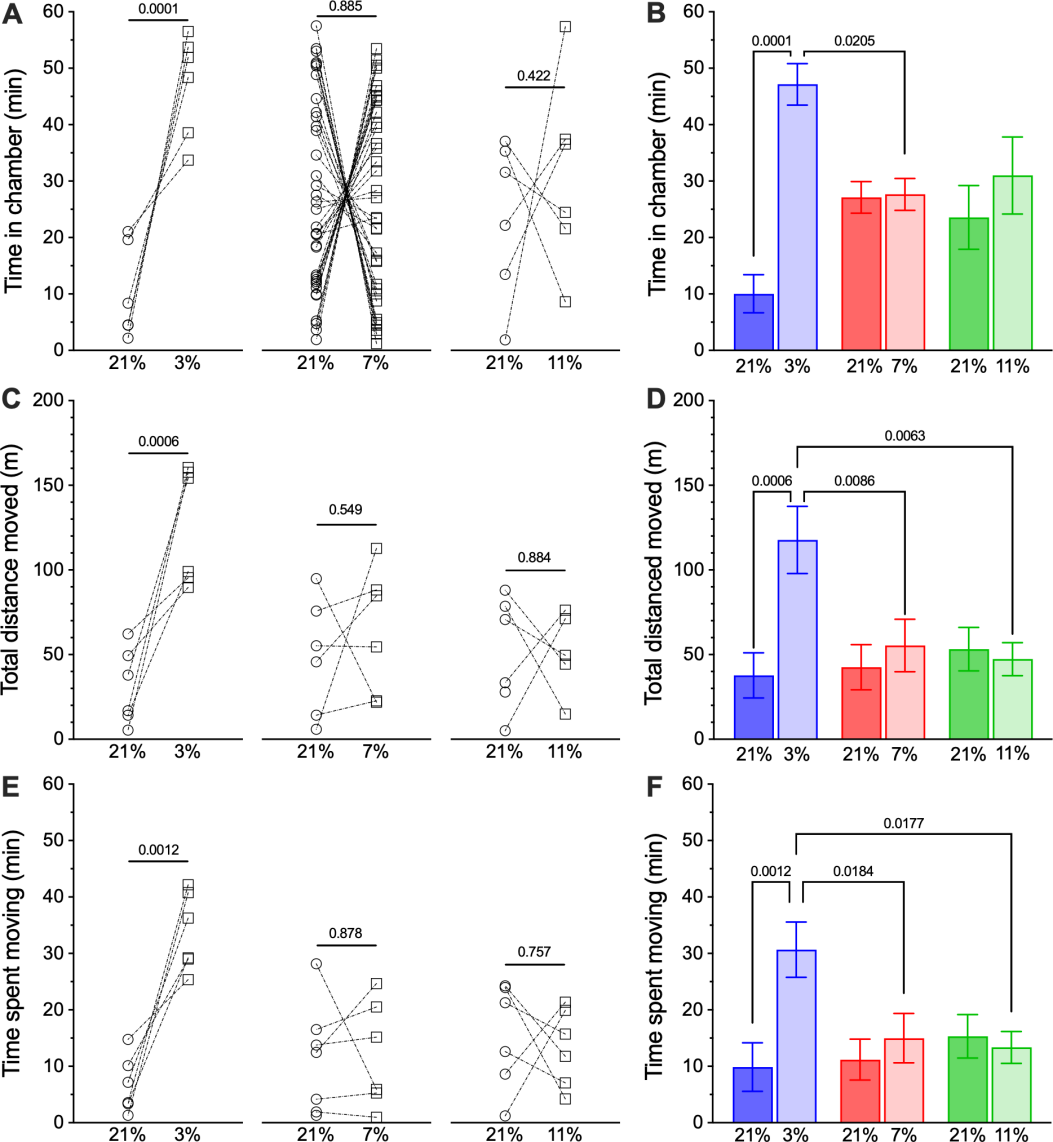
Solitary NMRs prefer severe hypoxia to normoxia. (A) Individual measurements of the time spent by solitary NMRs offered a choice between otherwise identical normoxic (21% O_2_; open circles) or hypoxic (11%, 7% or 3% O_2_; open squares) experimental chambers. (B) Mean ± s.e.m. for data from (A). (C) Individual measurements of the total distance travelled by individual NMRs in normoxic versus hypoxic chambers from (A). (D) Mean ± s.e.m. for data from (C). (E) Individual measurements of time spent actively moving by individual NMRs in normoxic versus hypoxic chambers from (A). (F) Mean ± s.e.m. for data from (E). Numbers indicate *p*-values derived from two-way analysis of variance (ANOVA) with Holm–Sidak post-tests, where *α* = 0.05.

We first evaluated the environmental preferences of individual NMRs. Analysis with a two-way ANOVA revealed a significant interaction between cumulation duration in a given chamber and the oxygen level (*F*_2,90_ = 6.72, *p* = 0.0019). Specifically, NMRs exhibited no behavioural preference for normoxia or hypoxia when given the option between normoxia and either mild hypoxia (figure 1A; 11% O_2_; 23.6 ± 5.7 min in normoxia versus 31.0 ± 6.8 min in hypoxia, *p* = 0.4225) or moderate hypoxia (7% O_2_; 27.1 ± 2.9 min in normoxia versus 27.6 ± 2.8 min in hypoxia, *p* = 0.8855). Conversely, solitary NMRs exhibited a strong behavioural preference for severe hypoxia (3% O_2_; 44.0 ± 5.9 min in hypoxia versus 13.2 ± 5.8 min in normoxia, *p* = 0.0001). Time spent preferentially in the 3% hypoxic chamber was also higher than the same variable in the 7% hypoxia group (*p* = 0.0205) but was not greater than the 11% hypoxic group (*p* = 0.635).

Similarly, when given the option between normoxia and severe hypoxia, NMRs moved a greater distance in the 3% O_2_ hypoxic chamber than the normoxic chamber (*F*_2,32_ = 4.66, *p* = 0.0167; figure 1B; 37.8 ± 13.3 m in normoxia versus 119.4 ± 18.5 m in hypoxia, *p* = 0.0006) and spent more time active in the hypoxic chamber than the normoxic chamber (*F*_2,32_ = 4.10, *p* = 0.0261; figure 1C; 9.9 ± 4.3 min in normoxia versus 30.7 ± 4.9 min in hypoxia, *p* = 0.0012). For both of these variables, values collected in the 3% hypoxia chamber were greater than the 7% or 11% hypoxia chambers (for distance moved in 3% versus 7% and 11%, *p* = 0.0086 and 0.0063, respectively; for cumulative duration moving in 3% versus 7% and 11%, *p* = 0.0184 and 0.0177, respectively). These differences are likely the result of the difference in time spent in the severe hypoxic chamber versus the normoxic chamber, because there was no difference in animal velocity between the chambers (see also below, data not shown). Conversely, there was no difference in distance moved or time spent active between the normoxic and hypoxic chambers in animals given the option between normoxia versus either moderate hypoxia (48.6 ± 14.1 m in normoxia versus 64.1 ± 15.2 m in hypoxia, *p* = 0.513 and 11.2 ± 3.6 min in normoxia versus 12.1 ± 3.9 min in hypoxia, *p* = 0.490, respectively), or mild hypoxia (50.6 ± 13.5 m in normoxia versus 51.3 ± 11.0 m in hypoxia, *p* = 0.780 and 15.3 ± 3.8 min in normoxia versus 13.4 ± 2.8 min in hypoxia, *p* = 0.739, respectively).

### 3.2. Social interaction shifts the depth of hypoxia that naked mole-rats prefer relative to normoxia

**Figure 2.**
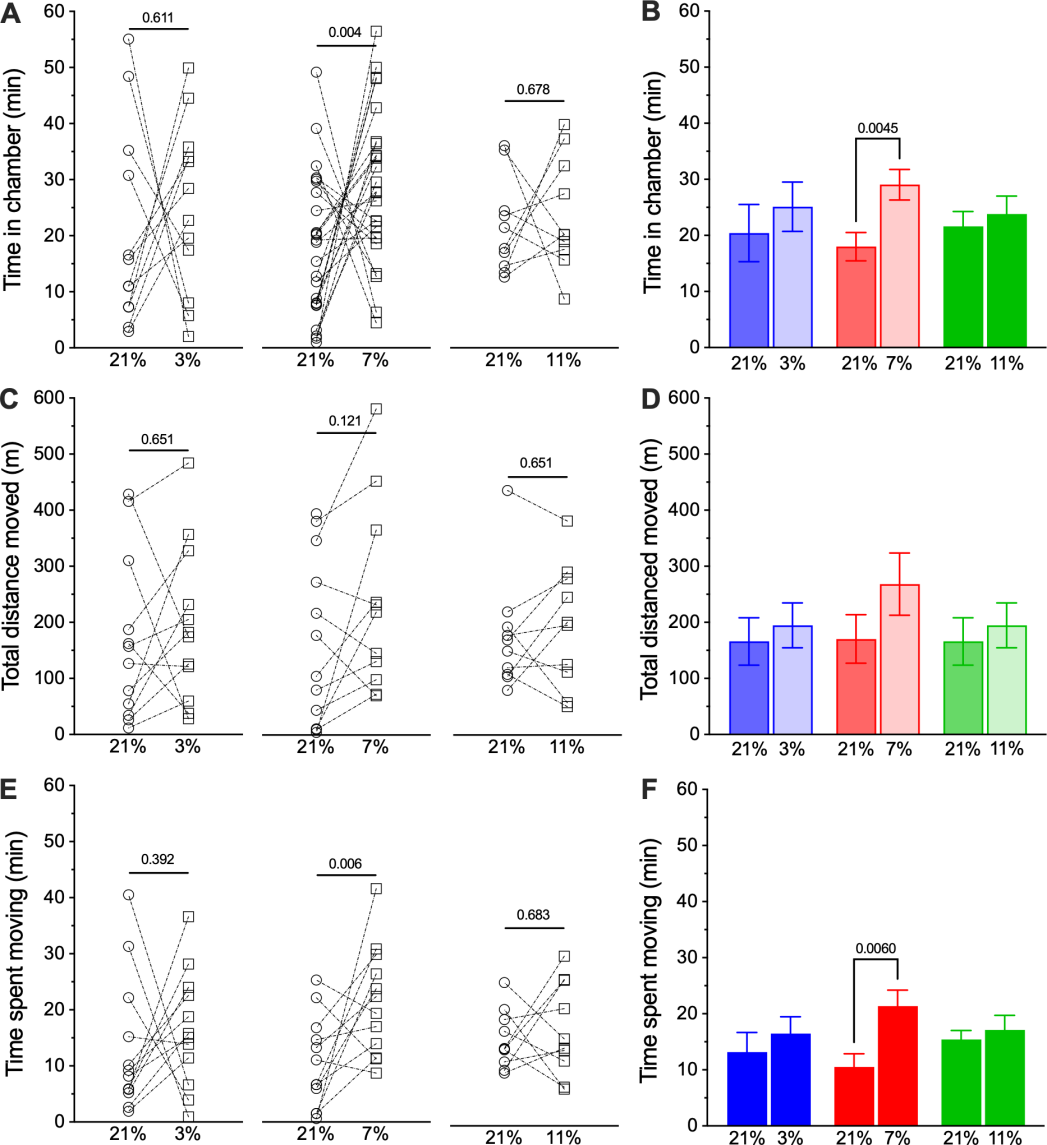
Paired NMRs prefer moderate hypoxia to normoxia. (A) Individual measurements of the time spent by pairs of NMRs offered a choice between otherwise identical normoxic (21% O_2_; open circles) or hypoxic (11%, 7% or 3% O_2_; open squares) experimental chambers. (B) Mean ± s.e.m. for data from (A). (C) Individual measurements of total distance travelled by pairs of NMRs in normoxic versus hypoxic chambers from (A). (D) Mean ± s.e.m. for data from (C). (E) Individual measurements time spent actively moving by pairs of NMRs in normoxic versus hypoxic chambers from (A). (F) Mean ± s.e.m. for data from (E). Numbers indicate *p*-values derived from two-way analysis of variance (ANOVA) with Holm–Sidak post-tests, where *α* = 0.05.

We have previously observed that social interactions alter behavioural and physiological responses to acute hypoxia in NMRs [21]; therefore, we next evaluated the environmental preferences of pairs of NMRs. Analysis with a two-way ANOVA revealed a significant impact on cumulation duration (*F*_1,88_ = 4.00, *p* = 0.0478). Interestingly, paired animals still preferred hypoxia to normoxia in some experiments, but the level of hypoxia at which this preference emerged shifted relative to results from individual animals. Specifically, paired animals had no behavioural preference for normoxia or hypoxia when given the option between normoxia and either mild hypoxia (figure 2A; 11% O_2_; 21.6 ± 2.7 min in normoxia versus 23.8 ± 3.2 min in hypoxia, *p* = 0.712) or severe hypoxia (3% O_2_; 20.4 ± 5.1 min in normoxia versus 25.1 ± 4.4 min in hypoxia, *p* = 0.391). Conversely, paired animals exhibited a strong behavioural preference for moderate hypoxia (7% O_2_; 18.0 ± 2.5 min in normoxia versus 29.1 ± 2.7 min in hypoxia, *t*_(48)_ = 2.985, *p* = 0.0045). Also, unlike in our solitary experiments, time spent preferentially in the 7% hypoxic chamber was not different from the same variable in the 3% or 11% hypoxia groups (*p* = 0.406 and 0.301, respectively).

In addition, when given the option between normoxia and moderate hypoxia, paired NMRs spent more time active in the hypoxic chamber than the normoxic chamber (figure 2C; 10.5 ± 2.3 min in normoxia versus 21.4 ± 2.8 min in hypoxia, *F*_1,62_ = 5.403, *p* = 0.0234), but did not move a greater distance in the 7% O_2_ hypoxic chamber than in the normoxic chamber (figure 2B; 170.5 ± 43.1 m in normoxia versus 268.4 ± 55.5 m in hypoxia, *F*_2,86_ = 0.414, *p* = 0.6628). For both of these variables, values collected in the 7% hypoxia chamber are not different from those collected in the 7% or 11% hypoxia chambers (for distance moved in 7% versus 3% and 11%, *p* = 0.5629; for cumulative duration moving in 7% versus 3% and 11%, *p* = 0.4059 and 0.3012, respectively). As with individual trials, the difference in time spent moving between chambers is likely the result of the difference in time spent in the moderate hypoxic chamber versus the normoxic chamber. Conversely, there was no difference in distance moved or time spent active between the normoxic and hypoxic chambers in animals given the option between normoxia versus either mild hypoxia (175.7 ± 32.1 m in normoxia versus 194 ± 34.1 m in hypoxia, *p* = 0.651 and 15.4 ± 1.6 min in normoxia versus 17.1 ± 2.6 min in hypoxia, *p* = 0.6, respectively), or severe hypoxia (166.2 ± 42.2 m in normoxia versus 194.6 ± 40.0 m in hypoxia, *p* = 0.392, and 13.2 ± 3.5 min in normoxia versus 16.5 ± 3.0 min in hypoxia, *p* = 0.291, respectively).

### 3.3. Sociality drives markedly increased activity in naked mole-rats

**Figure 3.**
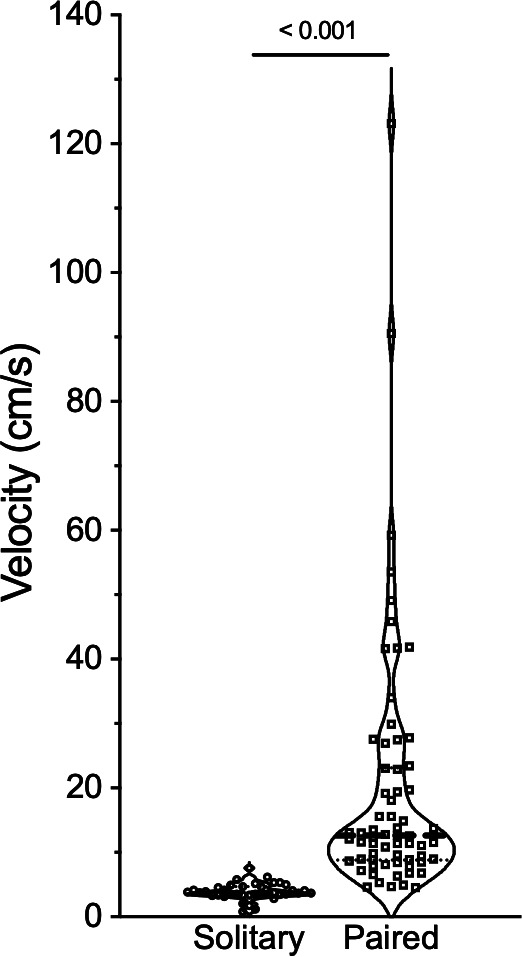
Sociality increases the velocity of active NMRs. Summary of the distribution of movement velocities from individual (open circles) and paired NMRs (open squares) across all treatment conditions. Data are presented as a violin plot with all data points shown. The thick dashed line indicates the median and the thin dotted lines indicate quartiles. Numbers indicate *p*-values derived from a Mann–Whitney test.

Pairs of animals were considerably more active than solitary animals, such that animals travelled approximately threefold to fourfold further during the duration of our experiments (figures 1B and 2B). Conversely, animals did not spend more time active as the total time spent moving between the solitary and paired experimental trials was similar (figures 1A and 2A). This increase in distance travelled was instead mediated entirely by animals in paired experiments moving at approximately fivefold greater velocity than animals in solitary experiments (figure 3; 3.9 ± 0.2 cm s^−1^ for solitary animals versus 19.8 ± 2.5 cm s^−1^ for paired animals; Mann–Whitney *U* = 46, *n*_1_ = 38, *n*_2_ = 66, *p* < 0.0001). Velocity did not differ between normoxic and hypoxic chambers in any experimental group (data not shown).

## 4. Discussion

We explored the hypothesis that hypoxia-tolerant NMRs exhibit hypoxic escape behaviours, prefer higher O_2_ environments, and that social interactions impact this behaviour. Surprisingly, we report that, when given the choice between normoxia and various levels of hypoxia, NMRs exhibit a normoxic escape response. Specifically, solitary NMRs prefer to spend more time in severe hypoxia than normoxia, with no apparent preference when offered a choice between mild or moderate hypoxia and normoxia. Conversely, pairs of NMRs prefer to spend more time in moderate hypoxia, presumably because markedly greater behavioural activity in a social setting increases energy demands and reduces the tolerability of severe hypoxia when animals are held in groups. Interestingly, animals in both solitary and social groups exhibit a high degree of variability in the choices made by individual animals, with some animals exhibiting strong preference for normoxia, some for hypoxia, and some expressing a weaker or no preference. This variability may be related to the personality of individual animals, which may interact with social cues to alter behavioural choices differently if the animal is alone versus in a group. To our knowledge, this is the first study to directly assay behavioural choice between normoxic and hypoxic environments in any mammal, or the impact of social interactions on these choices; therefore, complementary studies in other species, and particularly in non-eusocial species of mole-rat, would be of considerable value to the literature.

### 4.1. naked mole-rats exhibit a normoxic escape response

Contrary to our initial hypothesis, we report that individual NMRs behaviourally select for severe hypoxia when given the choice between this and a normoxic environment, but do not do so when offered a choice between mild or moderate hypoxia and normoxia. Furthermore, individual animals do not reduce behaviour (time spent active, distance travelled or movement velocity) in severe hypoxia. This is unlike the results of our previous studies, in which NMRs exposed to severe acute hypoxia, with no option to escape to normoxia, markedly reduced behavioural activity, including distance travelled and velocity [13]. Interestingly, a somewhat similar response has been reported in a study of moderately hypoxia-tolerant yellowtail kingfish (*Seriola lalandi*) [28]. Specifically, when these fish are exposed to escapable progressive hypoxia, they do not attempt to avoid hypoxia or exhibit behavioural or physiological stress. Instead, animals periodically enter well-oxygenated areas to reoxygenate, which seems to reduce the need for a hypoxic escape strategy.

The preference of NMRs for severe hypoxia is intriguing, but the underlying reason is unclear. It is possible that highly social NMRs are seeking conspecifics when they preferentially explore hypoxic chambers, as the most severely hypoxic region of their natural burrows is likely the densely populated nest chamber. Thus, severe hypoxia may act as an attractant. Alternatively, NMRs may be avoiding normoxia instead of seeking hypoxia as a sensory mechanism to detect fresh air and avoid burrow exit by solitary animals. Finally, NMRs may seek environmental means to reduce their metabolism as a behavioural preference. NMRs undergo robust metabolic rate suppression in severe hypoxia (approx. 85% in 3% O_2_ [15]) and hypoxia-tolerant animals often employ behavioural strategies that enhance metabolic rate suppression as a means of tolerating environmental hypoxia. For example, selecting a cooler environment increases an animal’s rate of heat loss, thereby lowering body temperature rapidly and conserving energy during hypoxia through systemic hypothermia [24,29]. This anapyrexia response widens the thermoneutral zone of an animal and induces a downward shift of its thermogenic threshold [30]. Small rodents such as rats, mice, hamsters and chipmunks exhibit behavioural thermoregulation when exposed to acute hypoxia [26,31], as do a variety of other species, including goldfish, lizards and toads [24]. NMRs do cease thermogenesis and reduce their body temperature to ambient levels in hypoxia [32,33], and also have access to cooler regions in deeper areas of their natural burrows (burrow temperature range at midday in peak summer: 24.6–48.8°C [17]). However, they do not behaviourally select for colder environments in hypoxia [22], unlike other small animals (although they prefer digging in colder soil in normoxia [17]). Instead, NMRs may behaviourally select for severe hypoxia to reduce their metabolic demand.

### 4.2. Sociality increases behavioural activity and shifts the naked mole-rat normoxic escape response to a less severe hypoxic threshold

Social interactions markedly increase the distance moved and movement velocity of NMRs, in both normoxia and hypoxia. This finding is consistent with a previous study exploring the impact of sociality on behavioural activity in either normoxia or acute hypoxia (7% O_2_), but wherein animals did not have the option to choose between gaseous environments [21]. Interestingly, the impact of sociality on overall behaviour is not affected by the gas composition of a given chamber, as there is no difference in velocity between normoxic and hypoxic regions in any of our solitary or paired experiments, whereas there is a large difference in velocity between solitary and paired experiments across treatments. This is important as other studies have noted that many species increase their activity levels in severe hypoxia, presumably as a last-ditch attempt to escape the environmental stress [28,34–36]. Thus, the absence of any effect of hypoxia on NMR velocity suggests an absence of hypoxia-induced stress-related behaviours. Instead, increased overall velocity in paired experiments is likely the result of social stimulus.

Importantly, increasing behavioural activity is energetically expensive in NMRs [37,38], and it is likely that increased activity driven by social interactions raises the demand for aerobic energy, which would in turn reduce an animals’ tolerance for environmental hypoxia. If this is the case, then severe hypoxia is likely less tolerable for pairs of NMRs than for solitary animals, and this may have shifted the preferred threshold for environmental hypoxia to a more moderate level than that preferred by solitary animals. Studies that evaluate the impact of sociality on behaviour-mediated metabolic demand would help to resolve this issue.

### 4.3. Conclusions

Our results demonstrate that subordinate NMRs employ an unexpected behavioural normoxic escape strategy when exposed to acute hypoxia, and that sociality impacts this strategy. We are not aware of any other species that chooses to ‘escape’ from normoxia to severe hypoxia. Furthermore, and unlike most animals, NMRs do not decrease activity in hypoxia if they can move intermittently to a normoxic environment. These behavioural findings likely reflect the lifestyle of these animals, which are thought to experience severe hypoxia in their communal nest chamber and may thus seek similar conditions in an experimental setting.

## Data Availability

Supporting data are available from the Dryad Digital Repository [39]. Supplementary material is available online [40].
